# A RasGAP, DAB2IP, regulates lipid droplet homeostasis by serving as GAP toward RAB40C

**DOI:** 10.18632/oncotarget.19960

**Published:** 2017-08-03

**Authors:** Xiaomin Luo, Chunman Li, Ran Tan, Xiaohui Xu, William K.K. Wu, Ayano Satoh, Tuanlao Wang, Sidney Yu

**Affiliations:** ^1^ Guangdong and Shenzhen Key Laboratory of Male Reproductive Medicine and Genetics, Institute of Urology, Peking University Shenzhen Hospital, Shenzhen PKU-HKUST Medical Center, Shenzhen, P.R. China; ^2^ School of Biomedical Sciences, The Chinese University of Hong Kong, Hong Kong SAR, P.R. China; ^3^ Department of Anesthesia, Faculty of Medicine, The Chinese University of Hong Kong, Hong Kong SAR, P.R. China; ^4^ School of Pharmaceutical Sciences, Xiamen University, Fujian, P.R. China; ^5^ The Graduate School of Natural Science and Technology, Okayama University, Okayama, Japan; ^6^ Epithelial Cell Biology Research Centre, The Chinese University of Hong Kong, Hong Kong SAR, P.R. China

**Keywords:** lipid droplets, RAB40C, GTPase activating protein, DAB2IP

## Abstract

Lipid droplet (LD) homeostasis involves activities of various RAB small GTPases. Recently, we found RAB40C was one of the RAB proteins regulating LD homeostasis. RAB40C contains a unique SOCS domain that is required for clustering of LDs. However, its precise functional role in LD homeostasis and mechanism of regulation remain largely unknown. In this study, we observed over-accumulation of LDs in cells with RAB40C deleted by Crispr-Cas9 editing. RAB40C appeared to reduce LD accumulation after long term incubation of cells with oleic acid (24 hours). Unexpectedly, we found that Ras GTPase activating protein (GAP), DAB2IP, bound to RAB40C mainly via its GAP domain and could serve as RAB40C GAP. Studies involving overexpression of DAB2IP and its GAP defective mutant and siRNA depletion of DAB2IP all confirmed that DAB2IP negatively regulated the effect of RAB40C on LD homeostasis. These results provide a novel perspective on the regulation of RAB40C and implicate various signalling pathways regulated by DAB2IP, which may play a role in LD homeostasis via RAB40C.

## INTRODUCTION

Excess neutral lipid molecules, such as triglycerides, sterol esters, and retinyl esters, are stored in lipid droplets (LDs) within hepatocytes and adipocytes [1]. In eukaryotes, LDs contain a central core of hydrophobic, neutral lipids covered with a monolayer of phospholipids bound by proteins. The protein components on the LD surface are biologically active and control the synthesis, storage and hydrolysis of the lipid contents and other LD-related cellular functions including trafficking of LDs and interactions with other organelles.

Proteomic analyses have identified proteins important in intracellular traffic such as small GTPases on LD surfaces [2, 3]. Among them, Rab18 was the best characterized Rab protein functioning on LDs [4-11]. Recently, RAB40C was reported to be LD-associated. [12]. Its expression increased as pre-adipocytes were differentiated to adipocytes. RAB40C was first cloned and characterized from an oligodendrocyte cDNA library [13]. The coding region of RAB40C contains 281 amino acids, longer than most small GTPases, because it contains a unique SOCS box domain between its conserved GTPase domain and its prenylated C-terminus. The SOCS box is important for the interaction with the Elongin B/C and Cul5 module [14, 15], and together they bind to RING finger proteins to form an active ubiquitin ligase [16] and to mediate a number of cellular processes [17, 18].

Although RAB40C was reported to mediate traffic in the perinuclear recycling compartment, recently we found that RAB40C was associated with the ER-Golgi intermediate compartment (ERGIC) and on the surface of LDs [12]. RAB40C formed homodimers via its SOCS box domain, and clustered LDs [12]. Although RAB40C localized on LDs and effected LD clustering, shRNA depletion of RAB40C resulted in only moderate change in LD accumulation. It is conceivable that the efficiency of RAB40C depletion might have contributed to a lack of stronger phenotype. LD morphology of cells completely deleted with RAB40C has not been determined, making it difficult to pinpoint the exact function of the regulation on RAB40C in LD homeostasis.

Many Rab proteins cycle between their active GTP-bound and inactive GDP-bound forms. RAB40C may have an additional layer of regulation due to the presence of a SOCS box. However, whether or not RAB40C is also regulated by the GTP/GDP cycle is a question remaining to be addressed. In this study, we present data to show that RAB40C regulates the accumulation of LDs in a guanine nucleotide dependent manner. Its activity could be negatively regulated by DAB2IP, a well-documented GAP protein for the Ras small GTPase. DAB2IP promotes the GTP hydrolysis of RAB40C almost as efficiently as that of H-Ras. These results provide a novel perspective on the regulation of Rab proteins by GAP proteins of the Ras sub-family and raise the possibility that signaling pathways may regulate LD homeostasis via RAB40C.

## RESULTS

### RAB40C regulates accumulation of LDs in nucleotide-dependent manner

**Figure 1:**
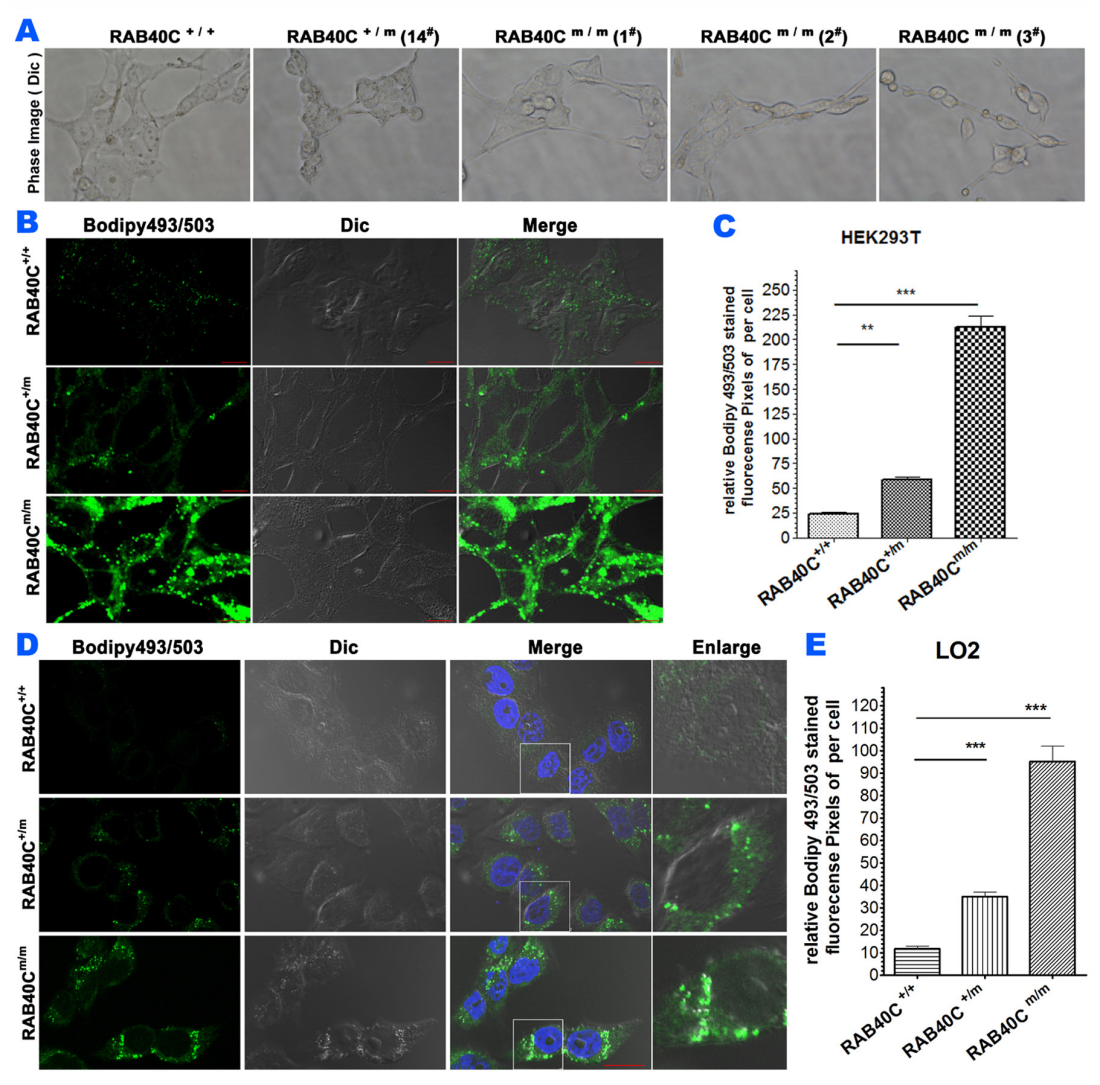
RAB40C deletion caused over-accumulation of lipid droplet The cells were fed with 40 μM oleic acid for 24 hours before fixation and staining. **(A)** Phase contrast images of wildtype HEK293T or various RAB40C mutant cell lines cultured in growth medium. **(B)** Bodipy493/503 staining in HEK293T cells of wildtype (Rab40^+/+^), RAB40C heterozygous (RAB40C^+/m^) and homozygous(RAB40C^m/m^) mutant cell lines. Scale bar = 10μm. **(C)** Quantified average fluorescent signals of Bodipy493/503 per cell in each sample in panel B. N≥36 per sample. Error bar = SEM. ** p < 0.01, *** p<0.001. **(D)** Bodipy493/503 staining in hepatocytesLO2 with the indicated genotypes with respect to RAB40C. Scale bar = 10μm. **(E)** Quantified average fluorescent signals of Bodipy493/503 per cell in each sample in panel D. N≥40 per sample. Error bar = SEM, *** p<0.001.

In order to study the function of RAB40C in vesicular transport and lipid droplet homeostasis, we prevented expression of RAB40C in HEK293T cells by Crispr-Cas9 genome editing. Detailed design and strategy for isolating RAB40C deleted cell clones can be found in Supplementary Figure 1. Organelle markers of the exocytic and endocytic pathways, including GM130, LAMP2, Sec31A, RAB5A, and ERGIC53 were not observed to be different between wild-type and RAB40C deleted cells (data not shown), suggesting RAB40C deletion did not affect the exocytic and endocytic pathways. However, it was obvious that small light-scattering droplets were present in the RAB40C^m/m^ (i.e. knockout) HEK293T cells, but not in wildtype (or RAB40C^+/+^) cells, when observed in a phase contrast microscope (Figure 1A). These structures were confirmed to be lipid droplets (LD) by staining cells with bodipy493/503,, a fluorescent dye that concentrates in neutral lipids (Figure 1B). Quantifications of bodipy493/503 fluorescence pixels per cell for each experimental group were measured and the degree of LD accumulation was correlated with the copies of RAB40C gene that had been edited. Cell containing heterozygous deletions in RAB40C (RAB40C^+/m^) accumulated LDs to an extent between wildtype and cells with homozygous deletions (Figure 1C). These results indicated that cells without functional RAB40C over-accumulated LDs. To determine if the effect of RAB40C in regulating lipid droplet homeostasis was different in cell types more relevant to lipid metabolism, we repeated RAB40C gene deletion in LO2 hepatocytes. This cell line originated from normal liver and contained LDs when cultured in growth medium (data not shown). When we serum-starved the cells before incubating with oleic acid of low dose (40 μM), we found that LO2 cells with RAB40C deleted over-accumulated LDs similar to HEK293T knockouts (Figure 1D–1E). In brief, these results indicated that RAB40C negatively regulated LD accumulation and its deletion led to over-accumulation of LDs.

**Figure 2:**
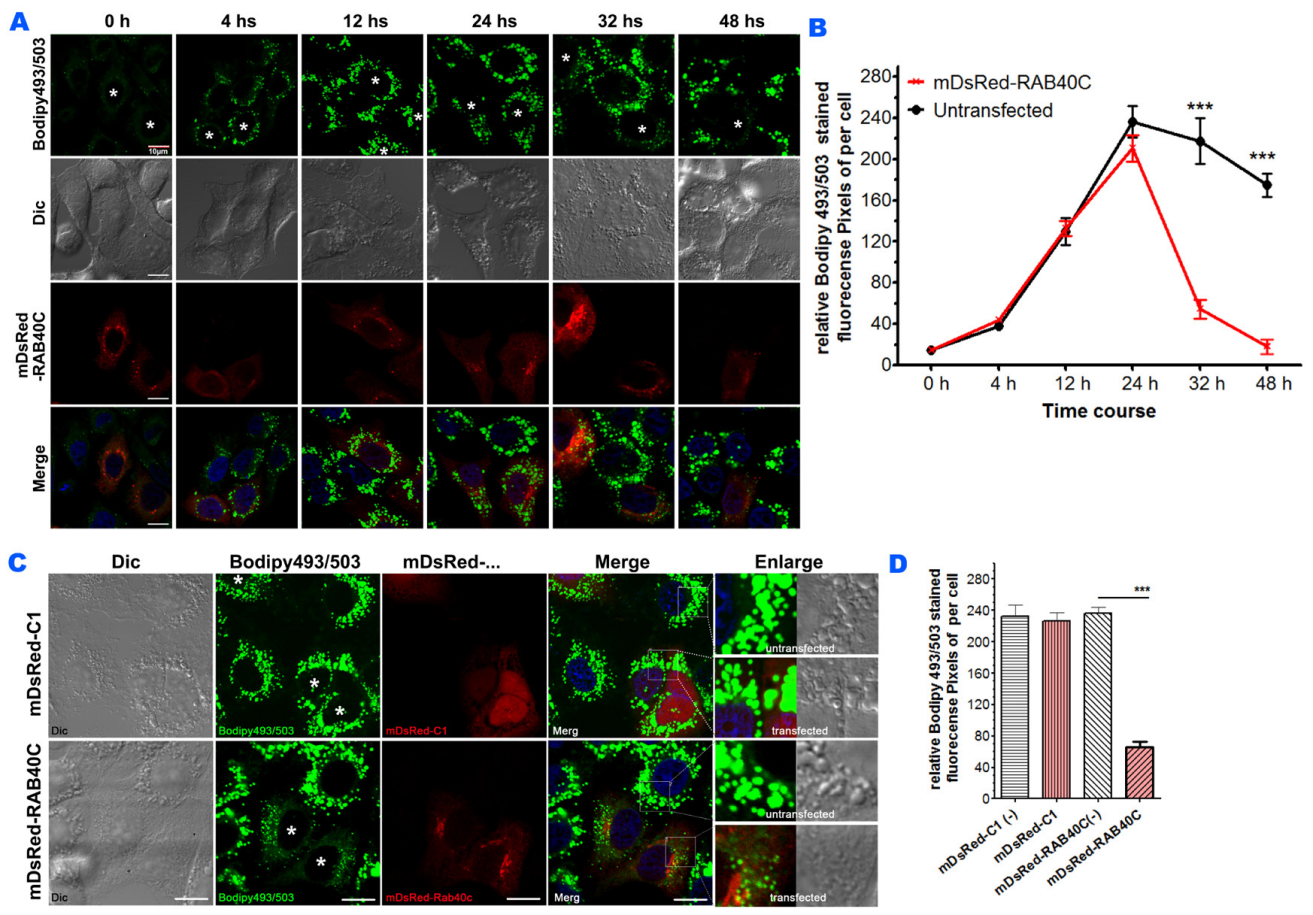
RAB40C reduced LD accumulation in LO2 cells **(A)** LO2 cells transfected with dsRed-RAB40C were incubated with oleic acid for the indicated time periods before fixed and staining with Bodipy493/503 for LDs. RAB40C transfected cells were marked with asterisks. Scale bar = 10 μm. **(B)** Quantification of the average Bodipy493/503 fluorescence signal per cell for the experiments shown in panel A. N ≥ 33. Error bars = SEM. *** p<0.001. **(C)** Bodipy493/503 staining of LO2 cells transfected with DsRed vector (top panels) or DsRed-RAB40C (bottom panels). **(D)** Quantification of the Bodipy493/503 fluorescence signal in DsRed vector or DsRed-RAB40C transfected and non-transfected cells. mDsRed-C1(-) indicates non-transfected cells in the sample transfected with DsRed vector. mDsRed-RAB40C(-) indicates non-transfected cells in the sample transfected with DsRed-RAB40C. N ≥ 30. Error bar = SEM. *** P< 0.001. In these experiments, the cells were fed with 400 μM OA for indicated time points in (A), and for 36 hours in (C).

As an alternative method to determine the negative regulation of LD accumulation by RAB40C, we overexpressed RAB40C in LO2 cells, loaded the cells with oleic acid and observed if LD accumulation was reduced in the RAB40C-expressing cells. In this experiment, we increased the concentration of oleic acid for incubation to 400 μM (high dose) to increase the dynamic range of the experiment. As shown in Figure 2, LD accumulation started to saturate at 24 hours after oleic acid loading, and overexpression of mDsRed-RAB40C did not cause much change during this time period (Figure 2A–2B). However, RAB40C overexpression caused drastic reduction of the LDs after 24 hours. We confirmed this effect was caused by RAB40C because mDsRed vector did not reduce LD accumulation (Figure 2D–2E).

**Figure 3:**
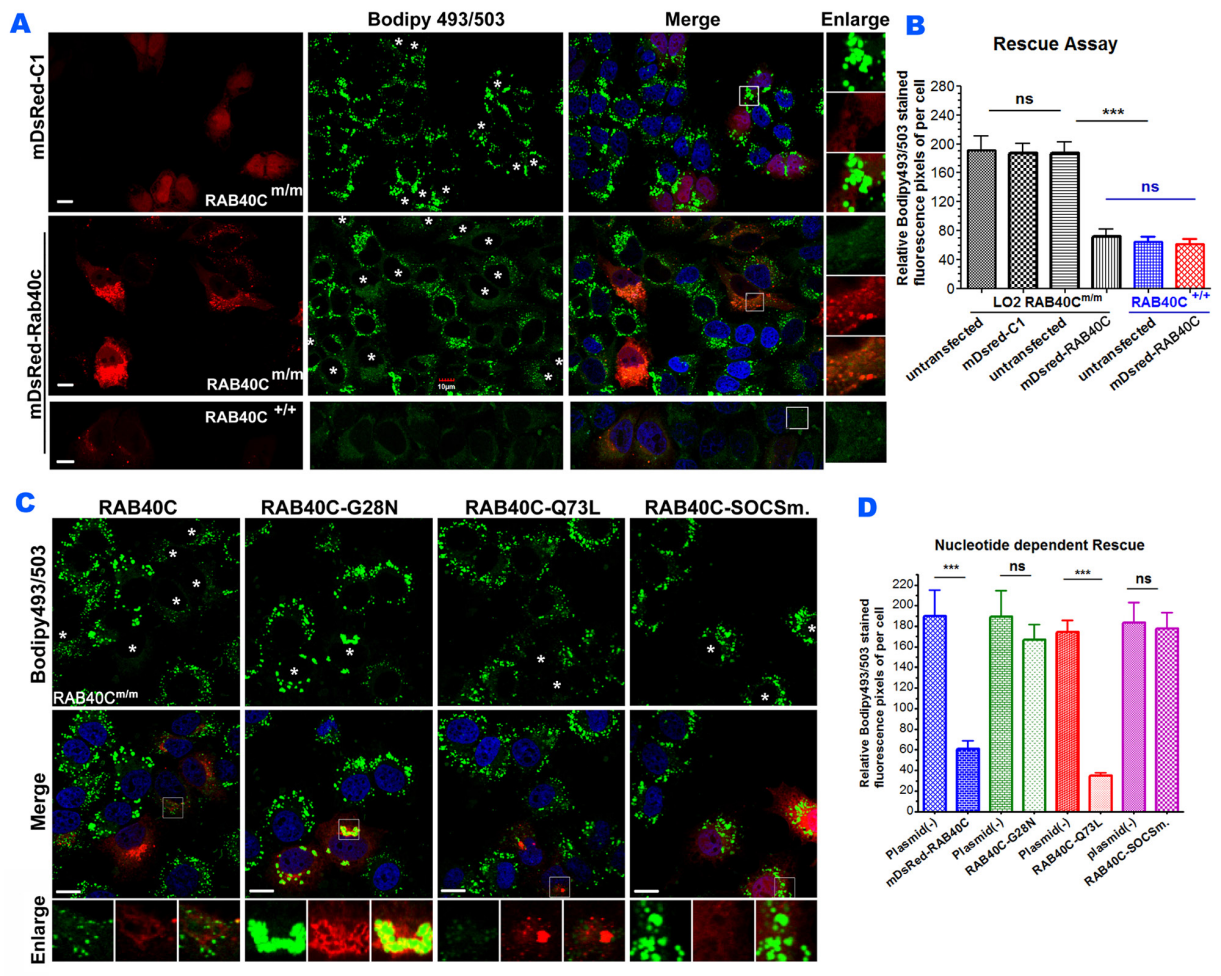
Re-introduction of RAB40C rescued the LD over-accumulation in RAB40C^m/m^ cells in a nucleotide-dependent manner **(A)** Bodipy493/503 staining in LO2 hepatocytes of the indicated genotypes transfected with mDsRed-RAB40C or mDsRed vector control. The cells were fed with 120 μM of oleic acid for 36 hours before fixation and staining. Transfected cells were indicated with asterisks. Scale bar = 10μm. **(B)** Quantified average fluorescent signals of Bodipy493/503 per cell in each sample in panel A. N≥30 per sample. Error bar = SEM. *** p<0.001. **(C)** Bodipy493/503 staining in RAB40C^m/m^ LO2 cells transfected with the wildtype DsRed-RAB40C, or GDP-locked (G28N), GTP-locked (Q73L), or SOCS mutant. Transfected cells were indicated with asterisks. Scale bar = 10 μm. **(D)** Quantified average fluorescent signals of Bodipy493/503 per cell in each sample. N ≥ 30 per sample. Error bar = SEM. *** p < 0.001.

If RAB40C deletion led to intracellular LD accumulation, re-introduction of RAB40C back to RAB40C^m/m^ cells should rescue or alleviate the LD accumulation. We transfected mDsRed-RAB40C into RAB40C^m/m^ LO2 hepatocytes and observed a drastic reduction of LD accumulation (Figure 3A). As a control, overexpression of mDsRed empty vector had no effect. Quantification of bodipy493/503 fluorescence confirmed that re-introducting RAB40C significantly reduced the accumulation of LDs (Figure 3B). To further investigate which nucleotide binding status of RAB40C was responsible for the increased accumulation of LD, we expressed various guanine nucleotide binding mutants of RAB40C in the RAB40C deleted cells and determined how these mutants might change the LD accumulation. As shown in Figure 3C–3D, only wild type RAB40C and constitutively active, GTP-locked mutant, RAB40C-Q73L, reduced the accumulation of LD in RAB40C^m/m^ cells, with the GTP-locked mutant causing the most reduction. In contrast, accumulation of LDs persisted and the LDs seemed to be clustered in the perinuclear region in cells expressing the GDP-locked mutant, RAB40C-G28N (second column and insets, Figure 3C). The SOCS mutant, RAB40C-SOCSm, was mislocalized as previously reported [12] and also was unable to reduce the accumulation of LDs. All together, these results established the role of RAB40C in regulating the accumulation of LD in a GTP-dependent manner and the process required an intact SOCS box.

### RAB40C interacts with Ras GAP DAB2IP

**Figure 4:**
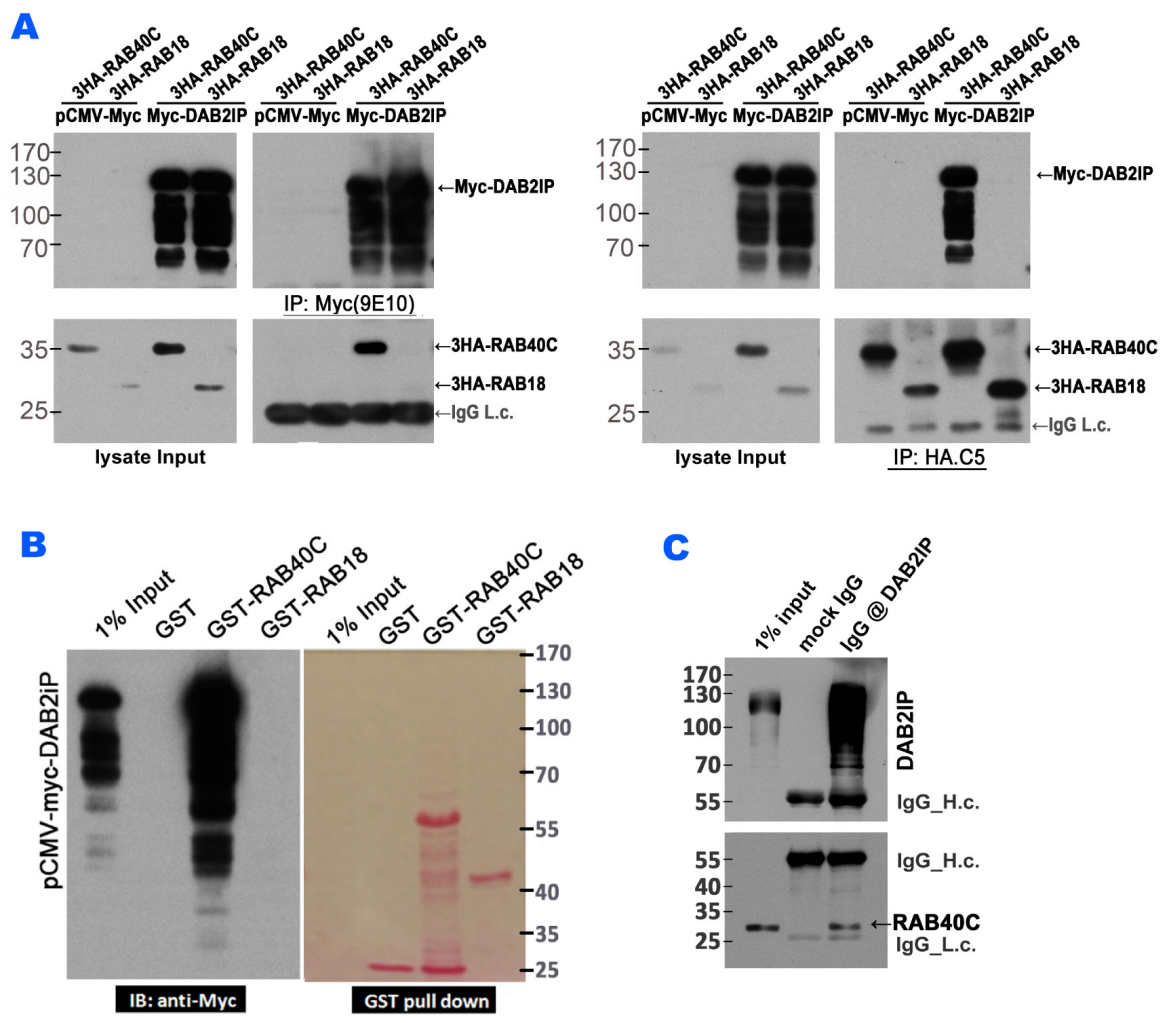
RAB40C interacted with DAB2IP **(A)** Myc-DAB2IP interacted with 3HA-RAB40C, but not with 3HA-RAB18. HEK293T cells transfected with the indicated expression plasmids were subjected to immunoprecipitation with anti-c-Myc (9E10) (left panels) or anti-HA (C5) (right panels), and the presence of co-purified proteins were detected by immunoblotting. **(B)** Lysates from HEK293T cells overexpessing Myc-DAB2IP were separately incubated with beads loaded with GST, GST-RAB40C and GST-RAB18. The presence of Myc-DAB2IP was detected by immunoblotting with anti-c-Myc antibody (left panel). Ponceau S staining of the same blot indicated the amount of GST proteins used for the pull down assay (right panel). **(C)** Immunoprecipitation of endogenous DAB2IP with anti-DAB2IP antibody also precipitated overexpressed RAB40C.

Since the guanine nucleotide binding status of RAB40C was important for its regulation of LD accumulation, we explored potential regulators, e.g., guanine nucleotide exchange factor (GEF) or GTPase-activating protein (GAP) for RAB40C by TAP-tagged purification of proteins bound to RAB40C followed identification of the bound proteins by mass spectrometry (data not shown). Rab GAPs have a consensus TBC domain, but we did not identify any RAB40C-binding proteins with this domain. However, DAB2IP, a Ras GAP, was precipitated with RAB40C and caught our attention. We performed co-IP and GST-pull down experiments to test if DAB2IP could interact with RAB40C. In a co-IP experiment that precipitated Myc-DAB2IP using anti-c-Myc(9E10) antibody, co-expressed HA-tagged RAB40C was detected in the precipitate (left panels, Figure 4A). As a control for this experiment, HA-tagged Rab18, a Rab also well-documented to regulate LD, did not co-precipitate with Myc-DAB2IP. When the co-IP experiment was performed reciprocally using anti-HA(C5) antibody for immunoprecipitation, Myc-DAB2IP was observed to co-precipitate with HA-RAB40C but not with HA-RAB18 (right panels, Figure 4A). Furthermore, when lysate from HEK293T cells overexpressing Myc-DAB2IP was subjected to GST-pulldown using recombinant GST-RAB40C purified from bacteria, Myc-DAB2IP was observed to bind to GST-RAB40C but not to GST-RAB18 (Figure 4B). We further determined if immunoprecipitation of endogenous DAB2IP could co-purify RAB40C. However, because endogenous RAB40C was difficult to detect, we needed to transfect RAB40C into cells to increase the level of expression. When we precipitated endogenous DAB2IP, we were able to detect co-precipitating RAB40C (Figure 4C). These experiments demonstrated that RAB40C physically interacted with DAB2IP.

Next, we mapped the domain(s) on DAB2IP that interacted with RAB40C. DAB2IP contains a PH-like domain followed by a C2 domain that are important for binding phospholipids, a RasGAP catalytic domain in the middle of the molecule, followed by a domain with unknown function (DUF) and a coiled-coil (CC) domain at the carboxyl terminus (Supplementary Figure 2A). We generated Myc-tagged fragments of DAB2IP with various combinations of DAB2IP domains and tested if they interacted with HA-RAB40C by co-IP experiments. As shown in Supplementary Figure 2B, fragments containing the GAP or CC domains consistently bound to RAB40C. Furthermore, each of these domains alone could bind to RAB40C (lanes 6 and 8, Supplementary Figure 2B) but with reduced affinity. Therefore, we concluded DAB2IP interacted with RAB40C via its RasGAP domain and the coiled-coil domain. The interaction between DAB2IP GAP domain and RAB40C was further verified in a GST pulldown experiment in which lysates from HEK293T cells overexpressing Myc-tagged DAB2IP fragments were subjected to binding with RAB40C and H-Ras (Supplementary Figure 2C). Fragments containing the GAP domain were retained by GST-RAB40C and GST-H-Ras to similar extent. We investigated the cellular location of the DAB2IP fragments co-expressed with RAB40C (Supplementary Figure 3). Many DAB2IP fragments affected the perinuclear localization of mDsRed-RAB40C to varying degrees, but the fragments GAP and GAP-C substantially co-localized with mDsRed-RAB40C (panels 6 and 10, Supplementary Figure 3) and clustered the RAB40C signal into a few and bright fluorescence puncta. However, the CC domain was largely found in the cell periphery (panel 8, Supplementary Figure 3), with little if any, colocalization with mDsRed-RAB40C. Other fragments containing the GAP domain, including full-length protein and N-GAP (panels 1 and 11, Supplementary Figure 3), did not show strong colocalization with mDsRed-Rab40C. We thought the subcellular localization of DAB2IP must be determined by multiple domains and therefore, even though full-length DAB2IP and N-GAP contained the RAB40C-interacting GAP domain, other domains present may prevent the protein from colocalizing with RAB40C.

### DAB2IP negatively regulates RAB40C functions in LD homeostasis

**Figure 5:**
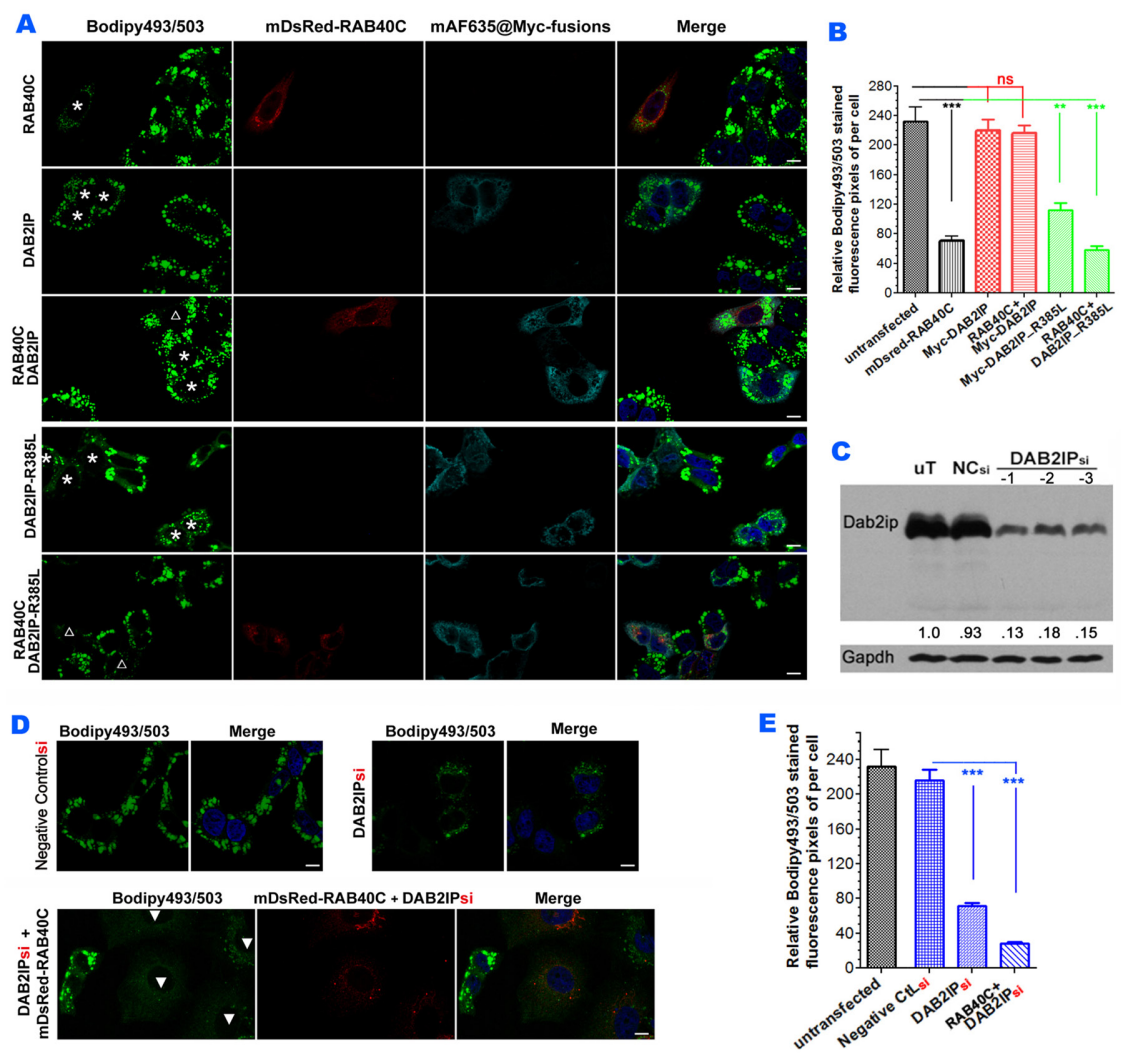
DAB2IP antagonizes RAB40C reduction of LD accumulation **(A)** Bodipy493/503 staining in hepatocytes LO2 overexpressed with mDsRed-RAB40C (top panel), Myc-DAB2IP (second panel), both RAB40C and DAB2IP (third panel), GAP defective DAB2IP mutant R385L (fourth panel), and co-expression with R385L and RAB40C (bottom panel). Cells expressing the indicated Myc-tagged fusion proteins are labeled with asterisks. Cells co-expressing both indicated proteins are labeled with empty triangles. The cells were fed with 400 μM of oleic acid for 36 hours before fixation and staining Scale bar = 10 um. **(B)** Quantification of Bodipy493/503 staining of samples shown in A. N ≥ 30. ** p < 0.01; *** p < 0.001. **(C)** siRNA depletion of DAB2IP in LO2 hepatocytes. Lysates from LO2 cells non-transfected (uT), or transfected with negative control siRNA (NCsi), or with siRNA duplexes specific to DAB2IP (DAB2IPsi were subjected to immunoblotting to determine the efficiency of DAB2IP knockdown. GAPDH serves as a loading control (bottom panel). **(D)** Bodipy493/503 staining in LO2 hepatocytes silenced with control siRNA (left two panels), or siRNA DAB2IPsi-1 (middle two), or DAB2IPsi-1 co-expressed with DsRed-RAB40C (right three). **(E)** Quantification of the Bodipy493/503 stainings of the samples shown in panel D. N ≥ 30. Error bar = SEM. ***P< 0.001.

To investigate if DAB2IP was involved in LD homeostasis mediated by RAB40C, we determined if DAB2IP was present on LDs. Immunofluorescence staining of DAB2IP showed that the protein was present on the plasma membrane and in intracellular membranes (Supplementary Figure 4A). DAB2IP signals in the cytoplasmic space in close proximity to LDs were readily observed at higher magnifications (Supplementary Figure 4B). To determine how DAB2IP might affect the function of RAB40C, we co-transfected DAB2IP and RAB40C into LO2 cells and investigated the effect on LD accumulation. As shown in Figure 5A, RAB40C overexpression reduced LD accumulation in LO2 cells and expression of DAB2IP alone did not affect LD accumulation (top two panels, Figure 5A). However, co-expression of DAB2IP and RAB40C (marked by empty triangle) prevented the reduction of LD accumulation caused by RAB40C alone (marked by asterisks). Co-expression of a catalytically defective mutant of DAB2IP, R385L, with RAB40C did not effect the activity of RAB40C in LD homeostasis (bottom panel, Figure 5A). Expression of R385L alone increased LD accumulation (fourth panel, Figure 5A), presumably because R385L might cause a dominant negative effect on endogenous DAB2IP. The R385L mutation was an amino acid change at one of the catalytic arginine residues critical for promoting GTP hydrolysis by Ras and mutating this residue caused partial loss of the GAP catalytic activity of DAB2IP [19, 20]. Statistical analysis of the bodipy493/503 fluorescence in each experimental condition in Figure 5A is presented in Figure 5B. To investigate whether endogenous DAB2IP also negatively regulated the function of RAB40C, we depleted DAB2IP in LO2 cells by siRNA. Transfecting siRNA duplexes for DAB2IP (indicated as DAB2IPsi) into cells reduced the protein expression of DAB2IP by 85 % (Figure 5C). DAB2IPsi, but not control RNAsi, reduced the accumulation of LDs similar to the R385L mutant (Figure 5D). LD accumulation almost vanished with overexpression of RAB40C (DsRed-RAB40C) in the DAB2IPsi depleted cells (solid triangles, Figure 5D), Statistical analysis of the quantifications of the relative bodipy 493/503 signals per cell are presented in Figure 5E. These results suggested that DAB2IP negatively regulated the effect of RAB40C on LD accumulation. More active RAB40C present in cells reduced LD accumulation when the function of DAB2IP was reduced by siRNA depletion or by overexpression of dominant negative R385L.

### DAB2IP served as GAP towards RAB40C

**Figure 6:**
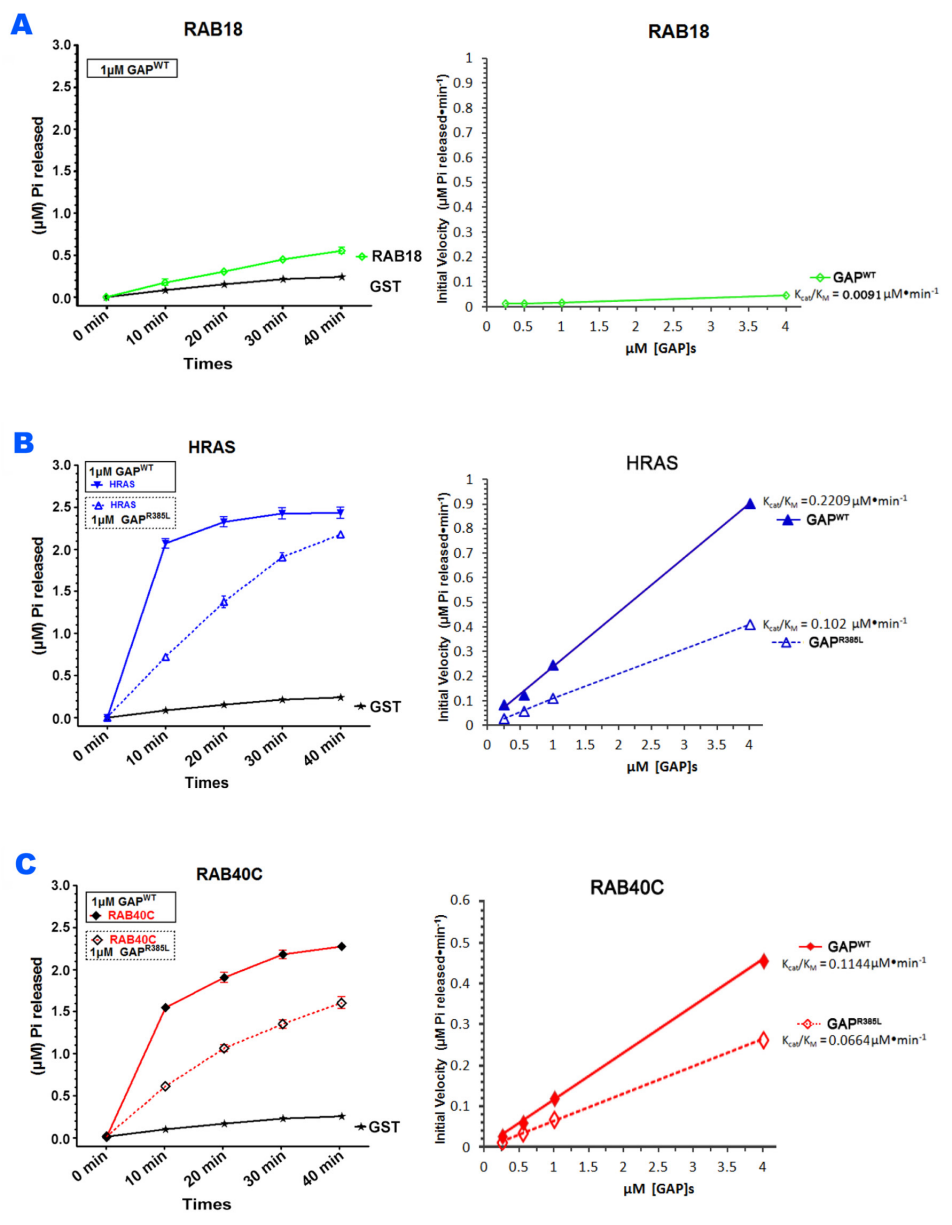
DAB2IP stimulated GTP hydrolysis of RAB40C **(A)** GAP domain of DAB2IP did not stimulate GTP hydrolysis of Rab18. GTP hydrolysis by GST-Rab18 (green line) was measured by the release of Pi as a function of time (left panel). GST (black line) was used as negative control. Catalytic efficiencies of DAB2IP toward Rab18 and GST were analyzed by Kcat/Km plot (right panel). **(B)** DAB2IP GAP domain stimulated GTP hydrolysis of H-Ras. GTP hydrolysis by GST-H-Ras was stimulated by wildtype GAP domain (solid blue line) or GAP defective mutant R385L (dotted blue line) as a function of time (left panel). Catalytic efficiencies of wildtype (solid blue line) and R385L (dotted blue line) GAP domains toward H-Ras were analyzed by K_cat_/K_M_ plot (right panel). **(C)** DAB2IP GAP domain stimulated GTP hydrolysis of RAB40C. GTP hydrolysis by GST-RAB40C was stimulated by wildtype GAP domain (solid blue line) or GAP defective mutant R385L (dotted blue line) as a function of time (left panel). Catalytic efficiencies of wildtype (solid blue line) and R385L (dashed blue line) GAP domains toward RAB40C were analyzed by K_cat_/K_M_ plot (right panel). All data points were averages of three independent experiments. Error bars = SEM.

Since DAB2IP had an inhibitory effect on the role of RAB40C in LD homeostasis and RAB40C directly bound to the GAP domain of DAB2IP, we suspected that DAB2IP served as a GAP for RAB40C. To test if DAB2IP could promote GTP hydrolysis of RAB40C, we purified various GST-fused recombinant proteins, including RAB40C, RAB18, H-RAS, and the wildtype and catalytically defective mutant of DAB2IP GAP domains (Supplementary Figure 5), and then carried out GAP assays. As shown in Figure 6A, the DAB2IP GAP domain did not stimulate generation of inorganic phosphate from GTP hydrolysis when GST or GST-Rab18 was used as substrates. As expected, when GST-H-RAS was used, DAB2IP GAP stimulated H-RAS GTP hydrolysis in a time and concentration dependent manner, whereas R385L mutant showed significantly reduced the GAP activity (Figure 6B). We performed this experiment with other concentrations of GAP (data not shown), and together the data allowed us to calculate the catalytic efficiencies (K_cat_/K_M_ ratio) by the GAP domains (right panels, Figure 6). We found that the DAB2IP GAP domain could promote GTP hydrolysis at 229 nM/min for H-RAS in this reaction setting (right panel, Figure 6B). When R385L mutant was used instead, the K_cat_/K_M_ ratio was reduced to 102 nM/min. Most importantly, DAB2IP also stimulated GTP hydrolysis of GST-RAB40C (Figure 6C), at a lower efficiency (114 nM/min) (right panel, Figure 6C). Again, when the R385L mutant GAP was used, the rate of GTP hydrolysis by RAB40C was reduced, with K_cat_/K_M_ ratio reduced to 66 nM/min. We obtained similar results when His-tagged recombinant proteins were used in the GAP assay, eliminating a potential artifact caused by GST-tag (data not shown). Together these results demonstrated that DAB2IP was a RAB40C GAP.

## DISCUSSION

In this paper, we showed that accumulation of LDs was negatively regulated by RAB40C. The effect of RAB40C on this process is dependent on its GTP binding and the integrity of its unique SOCS domain. We accidentally found that DAB2IP, a Ras GAP, was capable of serving as a RAB40C GAP and regulating the effect of RAB40C on the LD accumulation. The present study took advantage of the Crispr-Cas9 mediated deletion to generate a RAB40C null genetic background in HEK293T and LO2 cells. Unlike Huh-7 and HepG2 cells, which are cancer derived cell lines, LO2 are immortalized hepatocytes of non-cancer origin. The level of LDs is lower in LO2 than in Huh-7 and HepG2 but its capacity to form LDs remain similar to the other cell lines when fed with oleic acid. This feature allowed us to identify the effect of RAB40C as a negative regulator of LD accumulation (Figures 1–2). Overexpression of RAB40C did not affect the accumulation of LD during the first 24 hours of oleic acid incubation, but drastically reduced the LD contents at later time points. At present, we do not know why its effect of RAB40C appears only after 24 hours and what mechanism controls such delayed action, but it is tempting to hypothesize that RAB40C may protect against over accumulation of LDs in hepatocytes. Prolonged over-accumulation of LDs in hepatocytes leads to hepatic steatosis. The observation that DAB2IP acts as RAB40C GAP implies that the various signaling pathways that involve DAB2IP may also affect LD metabolism, with RAB40C being a likely converging point. DAB2IP is present on the plasma membrane and the cytoplasm. Such coordination may explain the delayed RAB40C action upon oleic acid incubation. The hepatocytes may secrete various cytokines, hormones and VLDL (very low-density lipoprotein) when incubated with oleic acid [21-24]. After 24 hours, these “immune response” cytokines and hormones act in autocrine fashion to reduce LD accumulation via RAB40C, presumably to prevent or reduce cytotoxicity associated with LD over-accumulation.

GAPs with dual specificity have previously been reported. In fact, SynGAP1, another member of same subfamily of Ras GAP as DAB2IP [25], stimulated the GTP hydrolysis by Rap more efficiently than Ras [26]. Another well-known Ras GAP, GAP1^IP4BP^, also acts as a Rap GAP [27]. DAB2IP has been reported to serve as Arf6 GAP and regulates TLR4-MyD88 signalling [28]. To our knowledge, this study is the first to report a Ras GAP also serving as a Rab GAP. Substrate degeneracy has long been observed in the TBC domain containing GAPs for Rab GTPases but most of the studies have been focused on members of the Rab subfamily of small GTPases. It is, therefore, very unexpected that DAB2IP can serve as a GAP with substrate specificity across three subfamilies of small GTPases, i.e., Arf, Rab and Ras. If DAB2IP has such low substrate specificity, one has to wonder how its GAP activity can be regulated so that the small GTPases can be properly regulated to control their respective cellular processes. We think other domains of DAB2IP may contribute to substrate specificity. An example for such mechanism can be found in the SynGAP1 C2 domain that is immediately amino-terminal to the GAP domain on the molecule. The SynGAP1 C2 domain stimulates the GAP activity toward Rap by four orders of magnitude [29]. While it is unlikely that the C2 domain of DAB2IP may also stimulate the GAP activity toward RAB40C by orders of magnitude, this domain and the PH domain on DAB2IP interact with phospholipids. It is conceivable that this domain may help localize the whole molecule to the phospholipid monolayer at the LD surface, so that DAB2IP may find access to LD-bound RAB40C.

DAB2IP is present on the plasma membrane and the cytoplasm. Shifting of DAB2IP from one intracellular compartment to another according to cellular changes may provide an additional layer of regulation for the small GTPase substrates that it helps inactivate. Of note, the relative abundance of DAB2IP was shifted (compared to wildtype cells) when lysate from RAB40C^m/m^ cells were fractionated in a sucrose gradient. Such result suggests that RAB40C somehow affects the intracellular localization of DAB2IP, and it is definitely possible that Arf6 and/or Ras activity may, in turn, be affected in RAB40C^m/m^ cells. While the precise relationship between DAB2IP and RAB40C, and their inter-relationships with Arf6 and Ras, await further investigation, this study warrants our reconsideration of how we perceive DAB2IP as a tumor suppressor and RAB40C as regulator of LD homeostasis, and how we classify GAPs with their small GTPases.

## MATERIALS AND METHODS

### Cell lines, cell culture reagents and transfection

Human Embryonic Kidney HEK293T (ATCC^®^ CRL-3216^™^) and human normal embryonic LO2 hepatocytes were cultured in high glucose Dulbecco’s Modified Eagle’s Medium (DMEM, Gibco^™^, #12800-017) with 10% fetal bovine serum (FBS, Sigma^™^, #A6003), 100 units/ml penicillin and 100 μg/ml streptomycin (Liquid Penicillin-Streptomycin, Gibco^™^, # 15140-122). Loading of oleic acid (OA) followed the method described previously [30]. Briefly, 40 to 120 μM OA (low dose) or 400μM (high dose) was added into LO2 hepatocytes at 16 hours after transfection experiments. Transfections were carried out with approximately 4×10^4^ adherent cells seeded on per cm^2^ surface in culture-plate(s) the night before, followed by transfection with 0.2 μg DNA per cm^2^ surface of cells at 60–80% confluence using jetPRIME® reagent (^#^114-75, Polyplus^®^-SA, France), according to the manufacturer’s instruction.

### Expression constructs

#### DNA constructs related to RAB40C

pEGFP-C1-RAB40C and the indicated mutants G28N, Q73L and SOCSm (LPLP_212-215_to AAAA) were constructed as previously described [12]. pNTAP-RAB40C, pmDsRed-RAB40C and mutant sequences containing G28N, Q73L and SOCSm were sub-cloned to pNTAP-A vector and pmDsRed-C1, respectively, using the restriction enzymes of EcoR I /Sal I. LentiCRISPRv2 (#52961) was purchased from Addgene^™^, and the lentiCRISPRv2-RAB40C_ gRNA1 & 2 were cloned by enzyme *BsmBI* according to lentiGuide oligo cloning protocol of the Zhang Lab [31].

### DNA constructs related to DAB2IP

The cDNA sequence of DAB2IP was purchased from PlasmID Repository (Harvard Medical School). The coding region was PCR-amplified and subcloned into mammalian expression vector pCMV-Myc between EcoR I and Sal I sites, to generate pCMV-myc-DAB2IP. The indicated deletion constructs for domain mapping experiments were all generated by PCR amplification of the desired fragment from DAB2IP cDNA and sub-cloned into the pCMV-Myc vector between the EcoR I and Sal I sites. The GAP catalytic deficient mutant (R385L), pCMV-myc-DAB2IP-R385L, was generated by PCR-directed mutagenesis approach. Primers used in generating these constructs are listed in Supplementary Table 1.

### Other DNA constructs

The mEGFP-H-RAS (#18662) was purchased from Addgene^™^. Bacterial expression constructs pGEX-4T3-RAB40C, pGEX-4T3-H-Ras, pGEX-4T2-Rab18, pGEX-4T2-GAP(containing 898-1884bp of DAB2IP), pGEX-4T2-GAPR^385L^, pYU-His-myc -RAB40C and pYU-His-myc-H-RAS (*abbr.* HM-RAB40C and HM-H-RAS) were all constructed by PCR and sub-cloning approach. All constructs were confirmed by DNA sequencing.

### Oligonucleotides

The primers used for plasmids construction and validation for cas9-mediated knockout were purchased from Tech-Dragon^™^ (Hong Kong SAR, China). siRNAs for depletion of DAB2IP were from purchased from GenePharma^™^ (Shanghai, China) (Supplementary Table 1).

### Antibodies

The Rabbit polyclonal antibody (pAb) against RAB40C was developed as previously described (Tan et al). The monoclonal antibody (mAb) against AIP-1 (F-3, ^#^sc-3565921) (also, DAB2IP) and against c-Myc (9E10, ^#^sc-40) were from SANTA CRUZ (Delaware Avenue, CA). mAb against HA-tag (C5^#^ AG-HT301) were obtained from Trans-Gene® (Beijing, China). Secondary antibodies conjugated with AlexaFluor488, AlexaFluor568 and AlexaFluor635 were purchased from Life Technologies^™^ (Thermo Fisher Scientific, Waltham, USA). HRP-conjugated secondary antibodies were purchased from Rockland (Philadelphia, USA).

### Cas9-mediated knock-out of RAB40C

Disruption of RAB40C gene in HEK293T and LO2 hepatocytes were adopted from the protocol of Dr. Ran [32]. Two guide RNAs targeting to the genomic locus of RAB40C (gRNA1 and gRNA2, Supplementary Table 1) were selected by DNA2.0 /CRISPR online tools. This guide RNA combination was introduced to cells to create two double stranded breaks (DSBs) around the first exon region (from -1000bp to +300bp) of RAB40C (Supplementary Figure 1). The transfection was carried out with an equimolar mixture of lentiCRISPRv2-RAB40C_gRNA1/2. 24 hours post-transfection, the cells were selected with 2 μg/ml puromycin for 3-7 days. At the end of the selection, the puromycin-resistant cells were trypsinized. Three quarter of the cells were subjected to genomic DNA isolation for evaluation of gene deletion efficiency. The remaining cells were replated in 96-well plates at density suitable for single colony isolation of the knockout cell clones. These clonal cell lines were picked from plates and were split into two new replica plates, with one plate for genomic DNA isolation and genotyping PCR, the other for cell expansion.

### Immunofluorescence microscopy

Cells grown on Nunc™ Thermanox™ coverslips (^#^174950, 12-mm) were fixed with 3.7% (v/v) paraformaldehyde for 20 min, and then permeabilized with PBS containing 1%BSA and 0.1%Saponin for 30 min. Afterwards, the cells were incubated with appropriate primary antibodies for 2 hours at room temperature. Primary antibodies were diluted to appropriate concentrations (usually 1 μg/ml) in PBS containing 1% BSA, except anti-DAB2IP (1 μg/ml in PBS + 5% BSA). After four washes, secondary antibodies conjugated with AlexaFluor-dye (Life Technologies^™^) were applied and incubated for 2 hours. Secondary antibodies were diluted 1000 fold from stock in PBS containing 1 % BSA. Samples were stained with the lipid-dye Bodipy493/503 (in 1:1000 dilution from stock, if needed to stain lipid droplets) and nuclear-dye DAPI for 30 min. The samples were mounted onto glass slides in Fluoromount-G (#0100-01, Southern BioTech™). Fluorescent signals were imaged using Olympus FluoView™ FV1200 confocal microscope and the captured images were analyzed by Olympus™ FV10-ASW RS4.1 software.

### Immunoprecipitation (IP)

Transfected HEK293T or LO2 cells in 10 cm dish(es) were lysed in RIPA buffer (20 mMTris pH 8.0, 100 mMNaCl, 0.1% NP-40 and 1× protease inhibitor cocktail Complete^®^ (^#^3RA04693132001, Roche™)) on ice. The cells were scraped and collected in 1.5 ml tubes. After brief vortex, the lysates were centrifuged at 14,000 rpm at 4°C for 15 minutes. The supernatants were subject to IP with 1–2 μg of anti-Myc or anti-HA antibody and 30 μl of protein-A sepharose slurry (^#^P3391, Sigma™). After 4 hours (overnight if necessary) of incubation at 4°C with end-to-end rotation, the immunoprecipitates were washed three times with lysis buffer and twice with cold PBS. The immunoprecipitates were eluted with 50μl of 2× SDS sample buffer and resolved on SDS-PAGE and analyzed by immunoblotting. For IP with endogenous DAB2IP, lysate generated from approximately 2×10^7^ HEK293T cells with overexpressed RAB40C were subjected to IP using antibody against DAB2IP, or control IgG against cyclin G1. The rest of the procedures are identical to the description stated above.

### Purification of recombinant proteins

pGEX-4T3-RAB40C, pGEX-4T3-H-RAS, pGEX-4T2-RAB18, pGEX-4T2-GAP, pGEX-4T2-GAP^R385L^, pYU-6×His-myc-RAB40C and pYU-6×His-myc-H-RAS were individually transformed into *E.coli* BL21(DE3). Fresh clones were picked from individual agar plates and amplified to large-scale growth (1 litre) at 37 C. After the culture reached to mid-log growth phase, protein expression was induced with 0.1 mM IPTG at 18 °C overnight. The bacterial cultures were harvested and resuspended in sonication buffer (50mM Tris PH8.0, 120mM NaCl and and 1× protease inhibitors) and the bacterial resuspensions were sonicated for a total of 3 minutes in 3 second pulses (Vibra Cell™, Kesheng Sonics, China). The homogenates were centrifuged at 20,000 g and the supernatants were loaded into a glutathione Agarose 4B resin column (for GST-fusions) or Ni-TA resin column (for His-fusions). After thorough washing, the fusion proteins were eluted from column by glutathione elution buffer (50mM Tris PH8.0, 10mM reduced glutathione) or by 400 mM imidazole in sonication buffer, respectively.

### GST pull down and related experiments

30 μl of GSH-4B resin loaded with GST, GST-RAB40C, GST-RAB18 and GST-GAP at concentration of approximately 1 mg/ml were incubated with lysates overexpressing the indicated proteins for 1 h at room temperature. Then the beads were washed for three times before subjected to analysis by immunoblotting.

### *In vitro* GAP assays

The procedure followed the principle developed by the laboratory of David Lambright [33], and instructions from the GTPase colorimetric assay kit (Cat. No. #602-0121, InnovaBiosciences®, Babraham, UK). Briefly, 40 μM of the indicated bacterially purified GST-fused small GTPases were loaded with 1 mM GTP in a 0.5 ml nucleotide loading buffer (20 mM Tris pH7.6, 150 mM NaCl, 5 mM EDTA, 1 mM DTT) for 3 hours at room temperature. Then, the excess nucleotide was removed by passing the reaction mixtures through Pierce Dextran D-slat columns (Thermo Scientific). The concentration of GTP-loaded small GTPases were determined and adjusted to a final concentration of 5 μM as a 2X substrate solution in column buffer (20 mM Tris pH7.6, 150 mM NaCl). Then a “2X GAP solution containing various indicated concentrations of of GST-GAP or GST-GAP^R385L^ mutant were made in Assay buffer (20 mM Tris pH7.6, 150 mM NaCl, 20 mM Mg_2_Cl). Release of inorganic phosphate (Pi) from GTP hydrolysis by the small GTPases was measured at various time points (i.e., 0, 10, 20, 30 and 40 min). Three experiments were performed for each data points. Then, the individual plots (n=3) containing the kinetic time courses for each small GTPase at the various GAP concentrations were generated in Prism 5.00.288 software, and initial velocity of reactions and *K*_*cat*_*/K*_*M*_ ratios were plotted by *v*_0_ /[small GTPase] versus [GAP] with a linear model: *F*_*t*_= *F*_0_+(*F*_*∞*_-*F*_*0*_) (1 - e^-Kobs t^).

### Statistical analysis

Unless indicated otherwise, all results were expressed as the mean ± SEM. Statistical analysis was performed with an analysis of variance (ANOVA) followed by the Dunnett’s Multiple Comparison Test analysis. P values less than 0.05 were considered statistically significant.

## SUPPLEMENTARY MATERIALS FIGURES AND TABLE




