# Correction: A RasGAP, DAB2IP, regulates lipid droplet homeostasis by serving as GAP toward RAB40C

**DOI:** 10.18632/oncotarget.24600

**Published:** 2018-03-02

**Authors:** Xiaomin Luo, Chunman Li, Ran Tan, Xiaohui Xu, William K.K. Wu, Ayano Satoh, Tuanlao Wang, Sidney Yu

**Affiliations:** ^1^ Guangdong and Shenzhen Key Laboratory of Male Reproductive Medicine and Genetics, Institute of Urology, Peking University Shenzhen Hospital, Shenzhen PKU-HKUST Medical Center, Shenzhen, P.R. China; ^2^ School of Biomedical Sciences, The Chinese University of Hong Kong, Hong Kong SAR, P.R. China; ^3^ School of Pharmaceutical Sciences, Xiamen University, Fujian, P.R. China; ^4^ Department of Anesthesia, Faculty of Medicine, The Chinese University of Hong Kong, Hong Kong SAR, P.R. China; ^5^ The Graduate School of Natural Science and Technology, Okayama University, Okayama, Japan; ^6^ Epithelial Cell Biology Research Centre, The Chinese University of Hong Kong, Hong Kong SAR, P.R. China

**This article has been corrected:** The proper order of the affiliations is as follows:

^**3**^ School of Pharmaceutical Sciences, Xiamen University, Fujian, P.R. China

^**4**^ Department of Anesthesia, Faculty of Medicine, The Chinese University of Hong Kong, Hong Kong SAR, P.R. China

Original article: Oncotarget. 2017; 8:85415-85427. https://doi.org/10.18632/oncotarget.19960

