# Supplementary material for: A RasGAP, DAB2IP, regulates lipid droplet homeostasis by serving as GAP toward RAB40C
Source: Oncotarget. 2017 Aug 3;8(49):85415–27. doi: 10.18632/oncotarget.19960 (PMC5689619; doi:10.18632/oncotarget.19960)
Supplement: Supplementary file 1 [file oncotarget-08-85415-s001.pdf]

A RasGAP, DAB2IP, regulates lipid droplet homeostasis by serving as GAP toward RAB40C

SUPPLEMENTARY MATERIALS

Supplementary Table 1: Primer sequence information.

See Supplementary File 1

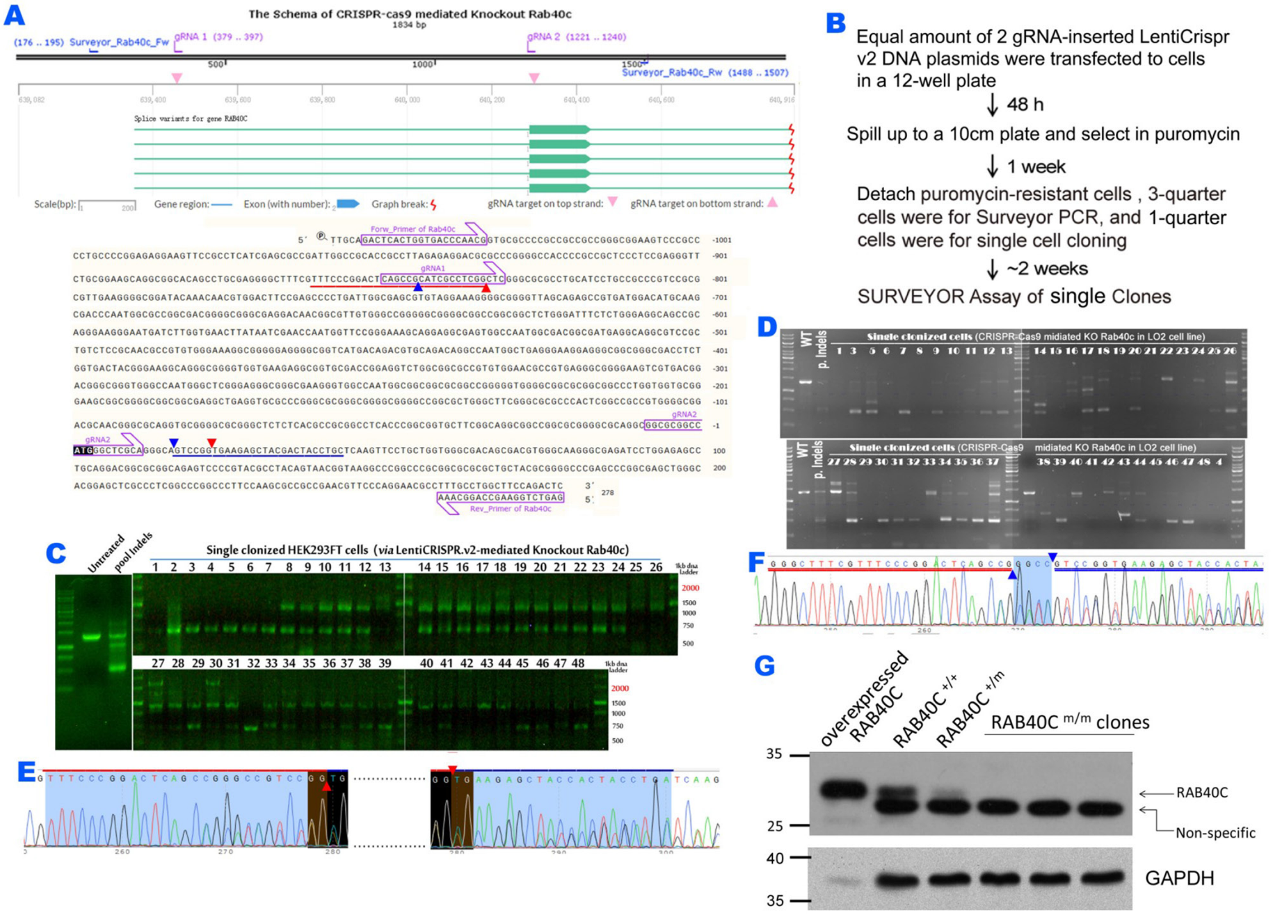

Supplementary Figure 1: Crispr-Cas9 mediated deletion of RAB40C. **(A)** Two guide RNAs encompassing approximately 840 base pairs (bp) within the RAB40C genomic locus were designed and intended to create indels in the first overlap region of all 5 variants of RAB40C. RAB40C specific Surveyor primers that flanked the knockout region were designed for subsequent genotyping. An example of indel from a RAB40C deleted HEK293T clone (sequence shown in E) was marked in the sequence (red arrowheads). An example of indel from Rab40C deleted LO2 clone (sequence shown in F) was marked as blue arrowheads. **(B)** Workflow of isolating RAB40C deleted cells. **(C)** Efficiency of deletion was tested with more than 50% efficiency (pool indels). Screening of HEK293T clones for RAB40C deletions. Genomic PCR of the RAB40C locus using the above Surveyor primers generated an approximately 1350 bp wildtype fragment. Any other PCR fragment size potentially contained indel in RAB40C locus. **(D)** Screening of LO2 hepatocytes for RAB40C deletions. **(E)** Sequencing example of a HEK293T clone. Deletion junctions were marked with red arrowheads and nucleotide position of the junction was indicated in A. **(F)** Sequencing example of a LO2 clone. Deletion junctions were marked with blue arrowheads and nucleotide position of the junction was indicated in A. **(G)** HEK293T cell lysates of the indicated genotypes were immunoblotted with anti-RAB40C (top) and GAPDH (bottom). All lanes were loaded with 36 µg of protein, except the lysate overexpressed with untagged RAB40C (5 µg was loaded, left most). Overexpressed, untagged RAB40C was run alongside to distinguish endogenous RAB40C from a prominent non-specific band of similar size.

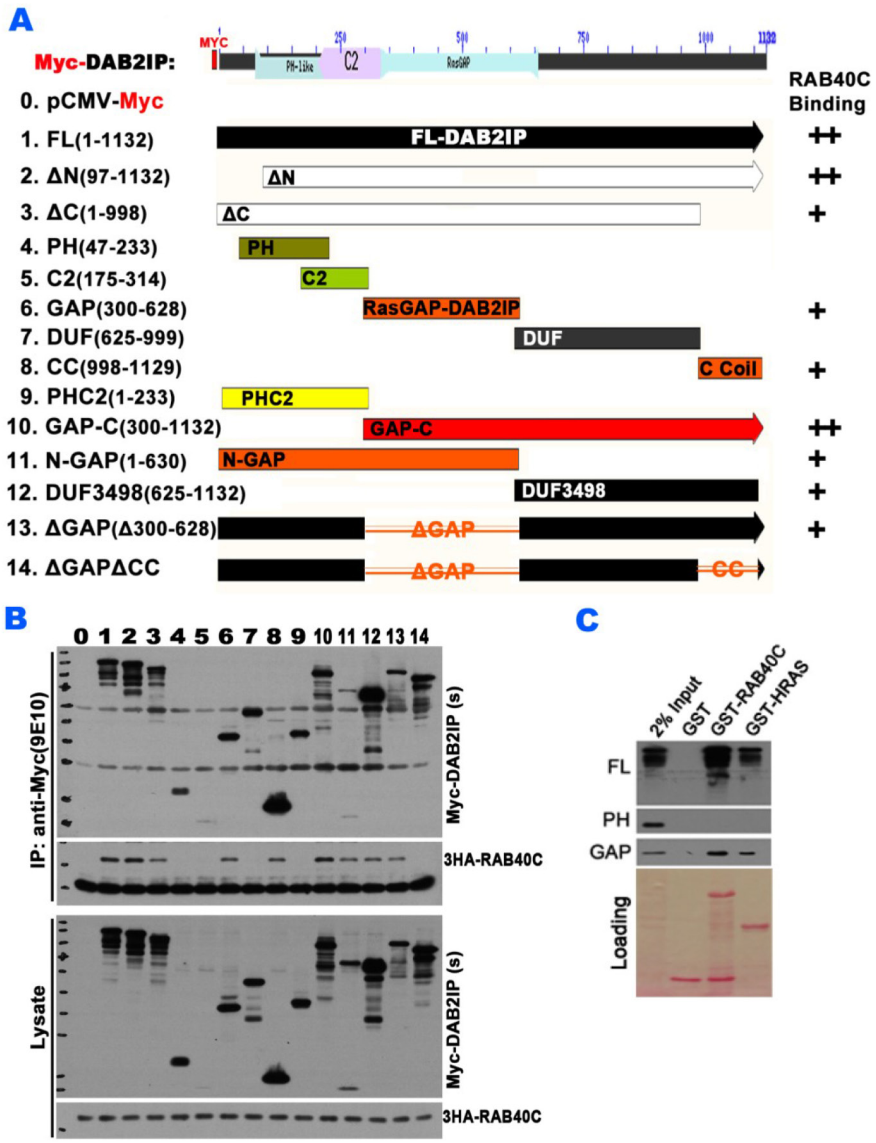

Supplementary Figure 2: Mapping of RAB40C-interacting domains on DAB2IP. **(A)** Schematic drawing of full-length human DAB2IP (NP_115941.2) and various deletions of the protein. Functional domains on DAB2IP are based on annotation by Simple Modular Architecture Research Tool (SMART) and the Conserved Domain Database by NCBI. **(B)** Various domains of DAB2IP in Myc-tagged expression vector were co-expressed with HA-Rab40C and tested for interaction by co-IP, using anti-c-Myc antibody. Expressions of the Myc-tagged DAB2IP fragments and HA-RAB40C (bottom two panels), the efficiencies of immunoprecipitation of the indicated Myc-tagged proteins (top panel), and the efficiency of HA-RAB40C co-precipitated (second panels) are shown. **(C)** Full-length (FL), PH domain (PH) and GAP domain (GAP) of DAB2IP in Myc-tagged were expressed in HEK293T cells. The cell lysates were subjected to pulldown by GST, GST-RAB40C and GST-HRas. The presence of various Myc-tagged DAB2IP fragment was probed with anti-c-Myc antibody (top three panels). Total protein was determined by Ponseau S staining of the blot (bottom panel).

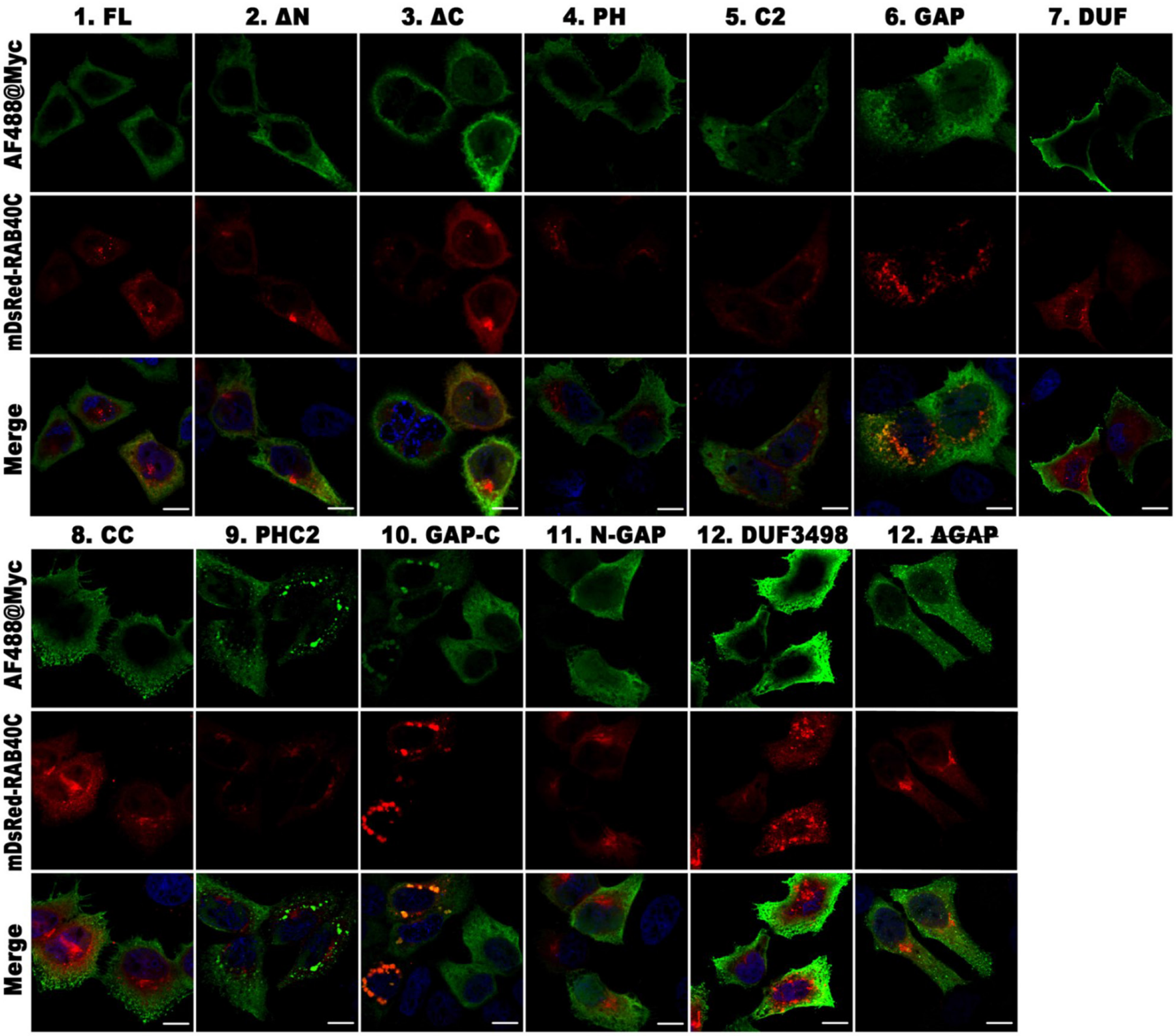

Supplementary Figure 3: Co-expression of various DAB2IP fragments with RAB40C. The indicated Myc-tagged DAB2IP fragments (green) and mDsRed-RAB40C (red) were co-transfected into LO2 cells before fixation and staining. Scale Bars = 10 μm.

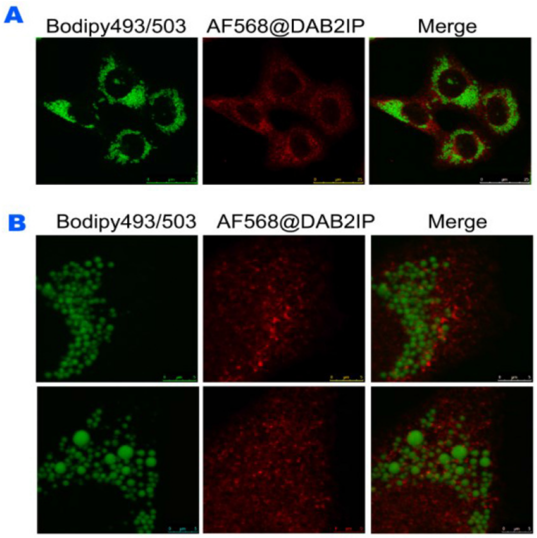

Supplementary Figure 4: Subcellular localization of DAB2IP by immunofluorescence staining. DAB2IP was stained with anti-DAP2IP antibody (red) and LD by Bodipy493/503. **(A)** The presence of DAB2IP at plasma membrane and internal punctate structures. Signal at the plasma membrane was indicated by arrowheads. Scale bar = 25 µm. **(B)** Higher magnification images of DAB2IP and LD staining. Scale bar = 5 µm.

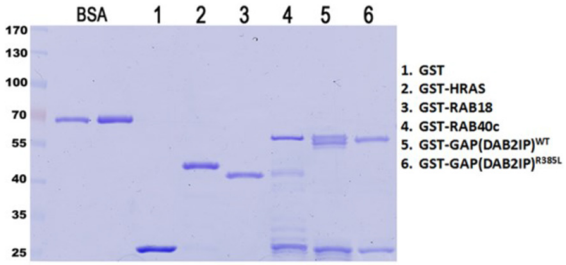

Supplementary Figure 5: Purified GST-fusion proteins for GAP assay. Estimated 1 µg of the indicated proteins (lanes 1-6) purified from bacterial overexpression was loaded onto SDS-PAGE for analysis. 0.5 and 1 µg of BSA were loaded onto the gel for comparison of protein quantity.
