# Supplementary material for: A RasGAP, DAB2IP, regulates lipid droplet homeostasis by serving as GAP toward RAB40C
Source: Oncotarget. 2017 Aug 3;8(49):85415–27. doi: 10.18632/oncotarget.19960 (PMC5689619; doi:10.18632/oncotarget.19960)
Supplement: Supplementary file 2 [file oncotarget-08-85415-s002.docx]

**Supplementary Table 1:** **Primer sequence information**

**DNA Oligos**

5'→3'

1

⑨RAB40C_gRNA1-F

CACCgccgcccgtccgcgcgttga

⑨RAB40C_gRNA1-R

AAACtcaacgcgcggacgggcggc

2

⑨RAB40C_gRNA2-F

CACCggcgcggccatgggctcgca

⑨RAB40C_gRNA2-R

AAACtgcgagcccatggccgcgcc

3

⑨RAB40C_Surveyor-F

GACTCACTGGTGACCCAACG

⑨RAB40C_Surveyor-R

GAGTCTGGAAGCCAGGCAAA

4

GST-RAB40C_EcoR1-F

GCGAATTCCCGGCTCGCAGGGCAGTCCGGTG

GST-RAB40C_Xho1-R

CCGCTCGAGCTAGGAGATCTTGCAGTTACT

5

Myc-FATP4_EcoR1-F

CGGAATTCGGATGCTGCTTGGAGCCTCTCT

Myc-FATP4_Xho1-R

CCGCTCGAGTCACAGCTTCTCCTCGCCTG

6

Myc-DAB2IP_EcoR1-F

TCGAATTCCCatgGAGACCCGACTCCCTTCTG

Myc-DAB2IP_Sal1-R

CGGTCGACCTAATGCATACTCTCTTTCAGCTG

7

3HA-RAB40C_EcoR1-F

CTGCAGAATTCCatgGGCTCGCAGGGCAGTCCG

3HA-RAB40C_Kpn1-R

AGCTTGGTACCGTCGACctaggagatcttgcagttactc

8

Myc-PH(Dab2ip)_EcoR1-F

ccGaattcCGttccgggtcacgggcttcc

Myc-PH(Dab2ip)_Sal1-R

tcgGTCGAccggttgggatgcaccgctcg

9

Myc-C2(Dab2ip)_EcoR1-F

ccGaattcACatcctgaagctgtgggtg

Myc-C2(Dab2ip)_Sal1-R

tcgGTCGAccacttctccacgaactgccg

10

Myc-GAP(Dab2ip)_EcoR1-F

ccGaattcAGggcggcaagggccctgg

Myc-GAP(Dab2ip)_Sal1-R

tcgGTCGAcCCgctacctggggtgctcag

11

Myc-DUF(Dab2ip)_EcoR1-F

ccGaattcCGagcgggcagctcccagggac

Myc-DUF(Dab2ip)_Sal1-R

tcgGTCGAcCTgtggggggggtctgggccca

12

Myc-CC(Dab2ip)_EcoR1-F

CCGaattcACagggataggctaaggag

Myc-CC(Dab2ip)_Sal1-R

TCGGTCGActctttcagctgggtcagggc

13

GST-HRAS_EcoR1-F

gCGAATTCCatgacggaatataagctggtgg

GST-HRAS_Sal1-R

tCGTCGACtcaggagagcacacacttgc

14

HM-RAB40C_Kpn1-F

tgGGTACC^CAGAAGCTGATCTCAGAGGAGGACCTG^GGCTCGCAGGGCAGTCCGGTG

HM-RAB40C_Sal1-R

agGTCGACCTAGGAGATCTTGCAGTTACTC

15

HM-hRAS_Kpn1-F

tgGGTACC^CAGAAGCTGATCTCAGAGGAGGACCTG^acggaatataagctggtggtg

HM-hRAS_Sal1-R

tCGTCGACtcaggagagcacacacttgc

16

sm_Myc-DAB2IP^-R385L^-F

cgagcacctcatcttcc**T**ggagaacacactggccaccaaggc

sm_Myc-DAB2IP^-R385L^-R

cagtgtgttctcc**A**ggaagatgaggtgctcgttgtccccgcagc

**RNA Oligos**

5'→3'

1'

DAB2IP_si_-1_sense

GCAGCAAGGAGGAAUACAUTT

……1_antisense

AUGUAUUCCUCCUUGCUGCTT

2'

DAB2IP_si_-2_sense

GGGAUAGGCUAAGGAGUAATT

……2_antisense

UUACUCCUUAGCCUAUCCCTT

3'

DAB2IP_si_-3_sense

CUGGAGCAGAGCAUAGUAUTT

……3_antisense

AUACUAUGCUCUGCUCCAGTT
